# Supplementary material for: Anti-Toxoplasma gondii Effects of Lipopeptide Derivatives of Lycosin-I
Source: Toxins (Basel). 2023 Jul 26;15(8):477. doi: 10.3390/toxins15080477 (PMC10467082; doi:10.3390/toxins15080477)
Supplement: Supplementary file 1 [file toxins-15-00477-s001.zip › toxins-2473333-supplementary.pdf]

Anti-Toxoplasma gondii Effects of Lipopeptide Derivatives of Lycosin-I

Xiaohua Liu, Peng Zhang, Yuan Liu, Jing Li, Dongqian Yang, Zhonghua Liu and Liping Jiang

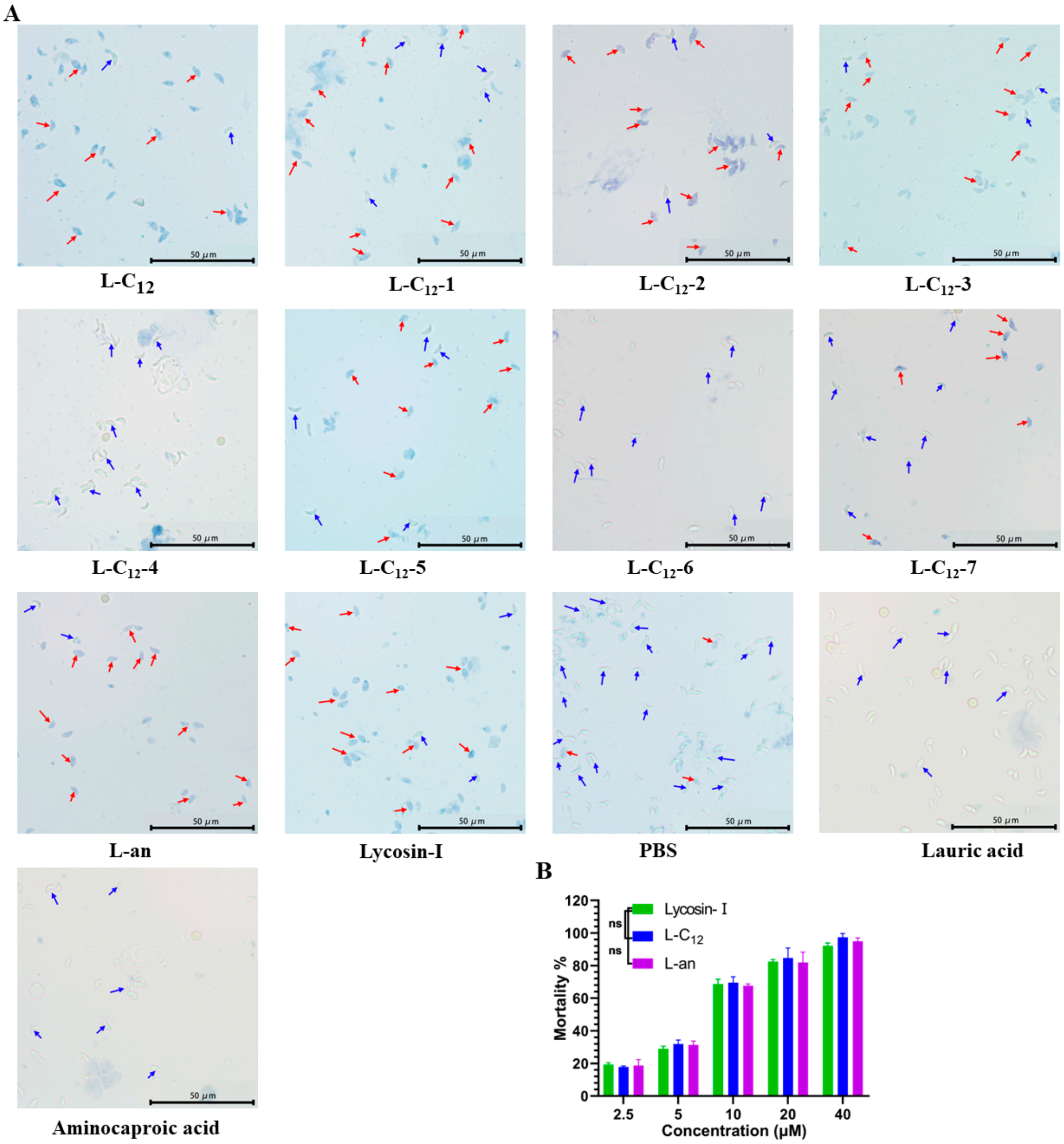

Figure S1. The effect of lipopeptides against *T. gondii* was evaluated by trypan blue assay. (A) Tachyzoites treated with control group (PBS) and lipopeptides at the concentration of 20 μM were observed by light microscopy. The red arrow points to the dead tachyzoites, while the blue arrow indicates live tachyzoites. (B) The tachyzoite viability was observed under a light microscope, and five fields were randomly selected to calculate tachyzoite mortality (ns > 0.05 in comparison with Lycosin-I).

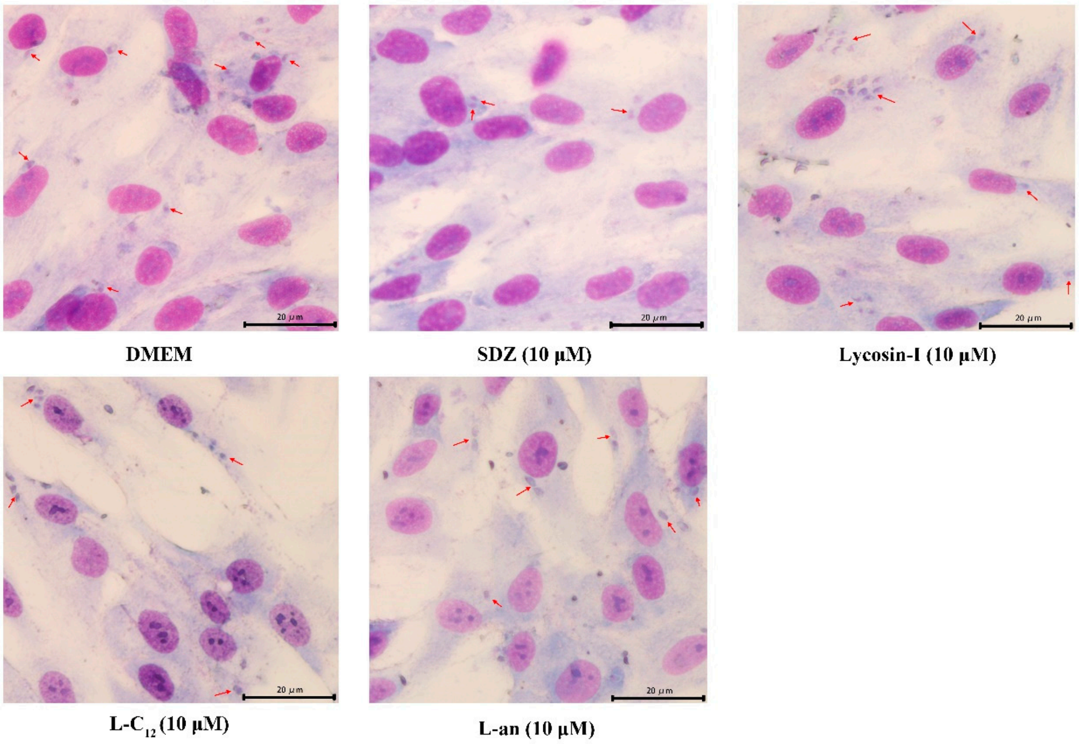

Figure S2. Effect of lipopeptides on tachyzoite invasion. Tachyzoites were pretreated with DMEM (negative control), SDZ (a clinical drug for toxoplasmosis, 10 μM), Lycosin-I (5 μM, 10 μM), L-C_{12} (5 μM, 10 μM), L-an (5 μM, 10 μM) before exposure to HFFs, respectively. HFFs in DMEM, SDZ, Lycosin-I (10 μM), L-C_{12}(10 μM), L-an (10 μM) were observed by a light microscopy (40×). Scale bars = 20 μm.

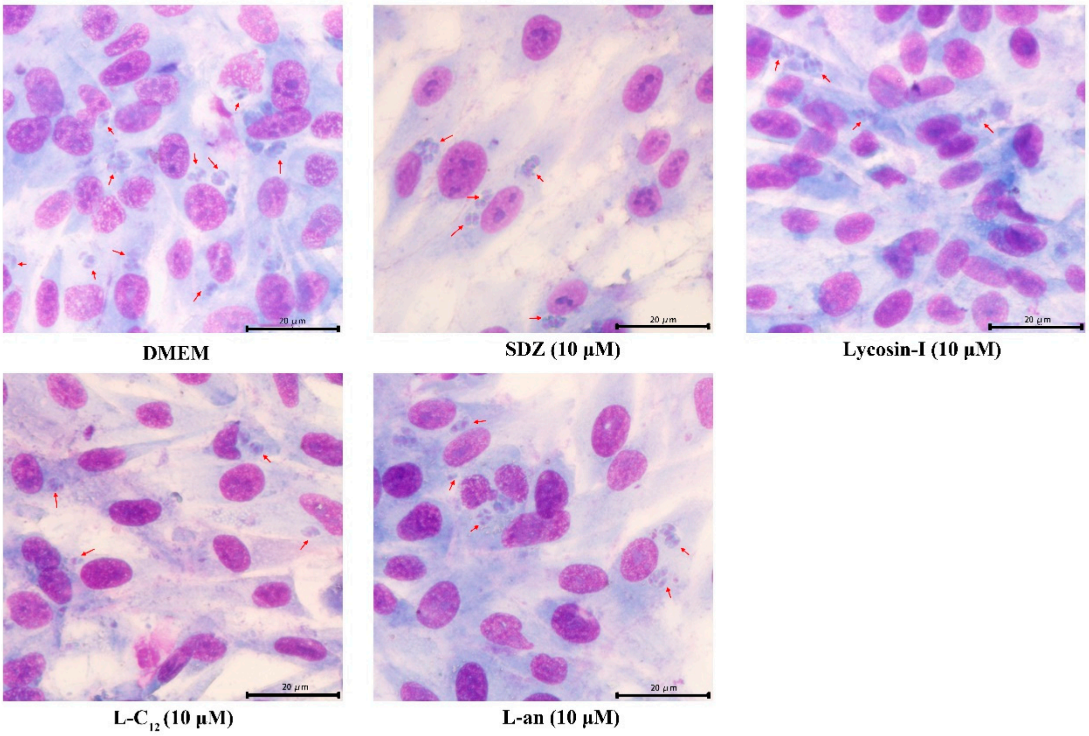

Figure S3. Effect of lipopeptides on tachyzoite proliferation. HFFs infected with tachyzoites were treated with DMEM (negative control), SDZ (a clinical drug for toxoplasmosis, 10 μM), Lycosin-I (5 μM, 10 μM), L-C_{12} (5 μM, 10 μM), L-an (5 μM, 10 μM) for 24 h, respectively. HFFs in DMEM, SDZ, Lycosin-I (10 μM), L-C_{12} (10 μM), L-an (10 μM) were observed by a light microscopy (40×). Scale bars = 20 μm.
